# Endoscopic hepaticogastrostomy combined with antegrade stent deployment using a multi-hole stent with a 5.9-Fr stent delivery system for patients with complete situs inversus

**DOI:** 10.1055/a-2625-3827

**Published:** 2025-07-04

**Authors:** Takeshi Ogura, Jun Matsuno, Takafumi Kanadani, Junichi Nakamura, Hiroki Nishikawa

**Affiliations:** 1 Endoscopy Center, Osaka Medical and Pharmaceutical University Hospital, Osaka, Japan; 2 2nd Department of Internal Medicine, Osaka Medical and Pharmaceutical University, Osaka, Japan

Endoscopic ultrasound-guided hepaticogastrostomy (EUS-HGS) is indicated for inaccessible papilla or isolated inaccessible biliary tract 1 2 3 . During endoscopic biliary drainage procedures, since the endosonography-created route (ESCR) might not form in patients complicated with ascites, bile leakage can occur as a complication in patients with high pressure in the biliary tract. In such cases, EUS-guided antegrade stent (EUS-AS) deployment might be helpful for decreasing the pressure in the biliary tract. However, in cases of antegrade stent deployment in the hepatic hilar region, bile duct branch obstruction can occur as a complication if a covered stent is deployed. To overcome this issue, a fully covered self-expandable metal stent with side holes (HANAROSTENT Biliary Multi-hole Benefit; M.I. Tech Co., Ltd., Pyeongtaek, South Korea) and a 5.9-Fr stent delivery system has become available (MHSEMS) ( Fig. 1 ). We herein report a case of EUS-HGS combined with EUS-AS using this stent in a patient with ascites and complete situs inversus.

**Fig. 1:**
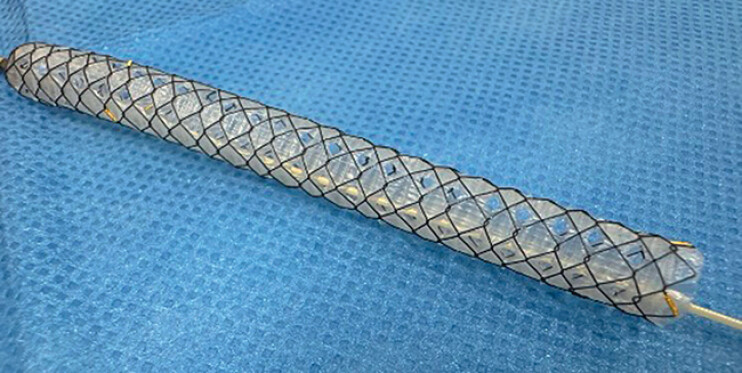
A fully covered self-expandable metal stent with side holes (HANAROSTENT Biliary Multi-hole Benefit; M.I. Tech Co., Ltd., Pyeongtaek, South Korea) and a 5.9-Fr stent delivery system.

A 77-year-old female was admitted for obstructive jaundice caused by a hepatic hilar cholangiocarcinoma. She had complete situs inversus. Although plastic stent deployment was successfully performed using a transpapillary approach, guidewire insertion into the left bile duct failed. Therefore, EUS-HGS was attempted. EUS imaging revealed the presence of ascites ( Fig. 2 ). First, the intrahepatic bile duct was punctured using a 19-G needle, and a contrast medium was injected. Then, a 0.025-inch guidewire was successfully inserted into the common bile duct across the obstructed site. Subsequently, a double lumen dilator was inserted, and an additional guidewire was deployed ( Fig. 3 ). The 5.9-Fr stent delivery system for the MHSEMS was easily inserted, and the MHSEMS was successfully deployed from the common bile duct to the left hepatic bile duct across the obstruction site ( Fig. 4 ). Finally, using a partially covered SEMS, EUS-HGS was performed without any adverse events. Cholangiography performed immediately after the procedure confirmed sufficient stent expansion ( Fig. 5 , Video 1 ).

**Fig. 2:**
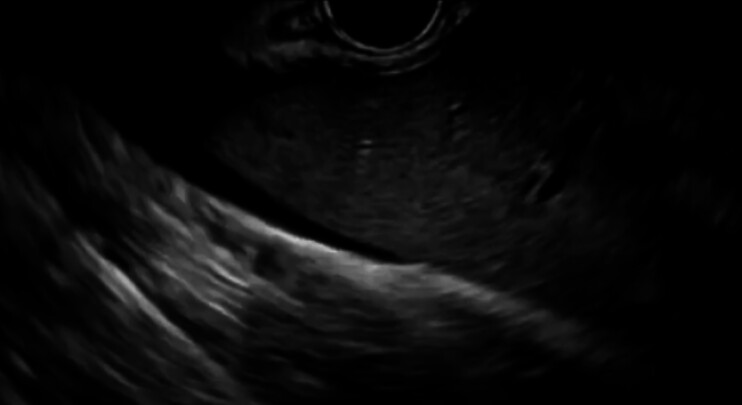
EUS imaging reveals the presence of ascites.

**Fig. 3:**
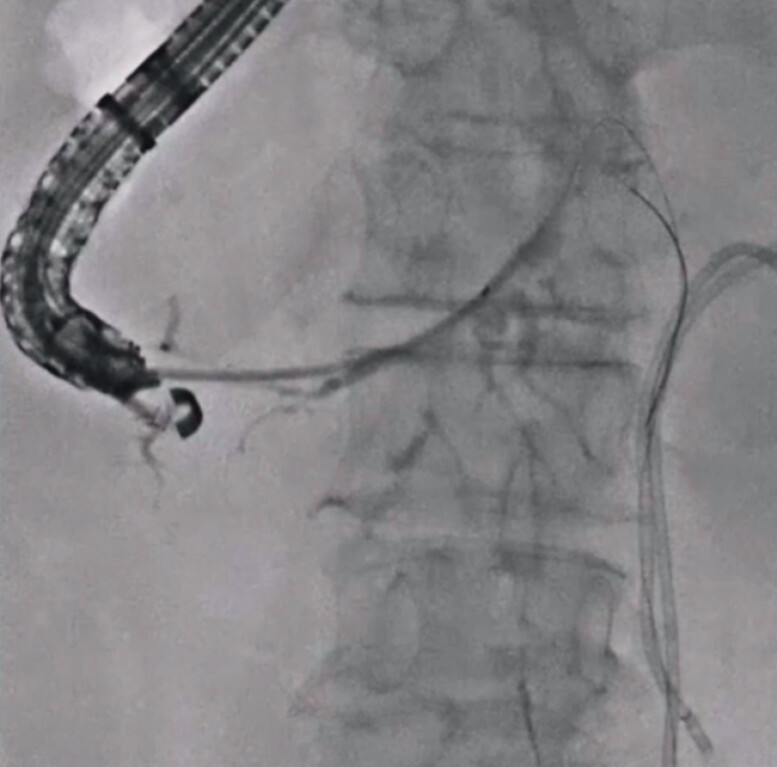
A double-lumen dilator is inserted, and an additional guidewire is deployed.

**Fig. 4:**
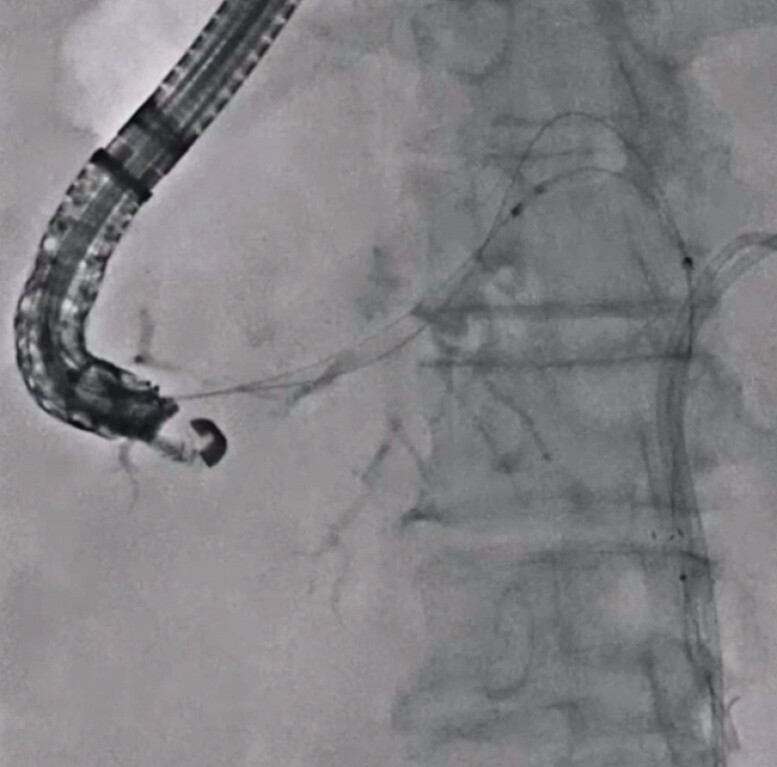
The multi-hole self-expandable metal stent is successfully deployed from the common bile duct to the left hepatic bile duct across the obstruction site.

**Fig. 5:**
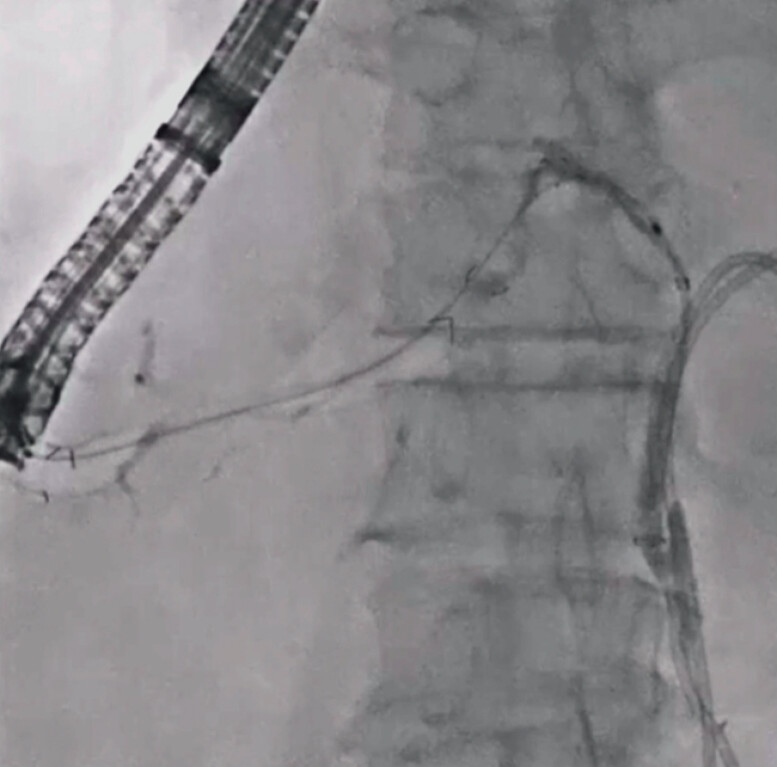
Cholangiography performed immediately after the procedure confirmed sufficient stent expansion.

The stent delivery system of the multi-hole self-expandable metal stent is easily inserted into the left hepatic bile duct.Video 1

In conclusion, EUS-AS using the MHSEMS might be useful for preventing bile duct branch obstruction and allows sufficient stent expansion immediately after the procedure. To the best of our knowledge, although EUS-HGS for complete situs inversus has been previously described, this is the first case report of EUS-HGS combined with the use of an EUS-AS.

Endoscopy_UCTN_Code_TTT_1AS_2AH

## Notes

Endoscopy E-Videos https://eref.thieme.de/e-videos
*E-Videos* is an open access online section of the journal *Endoscopy* , reporting on interesting cases and new techniques in gastroenterological endoscopy. All papers include a high-quality video and are published with a Creative Commons CC-BY license. Endoscopy E-Videos qualify for HINARI discounts and waivers and eligibility is automatically checked during the submission process. We grant 100% waivers to articles whose corresponding authors are based in Group A countries and 50% waivers to those who are based in Group B countries as classified by Research4Life (see: https://www.research4life.org/access/eligibility/ ). This section has its own submission website at https://mc.manuscriptcentral.com/e-videos .
